# Dmrt5, a Novel Neurogenic Factor, Reciprocally Regulates Lhx2 to Control the Neuron–Glia Cell-Fate Switch in the Developing Hippocampus

**DOI:** 10.1523/JNEUROSCI.1535-17.2017

**Published:** 2017-11-15

**Authors:** Bhavana Muralidharan, Marc Keruzore, Saurabh J. Pradhan, Basabdatta Roy, Ashwin S. Shetty, Veena Kinare, Leora D'Souza, Upasana Maheshwari, Krishanpal Karmodiya, Agasthya Suresh, Sanjeev Galande, Eric J. Bellefroid, Shubha Tole

**Affiliations:** ^1^Department of Biological Sciences, Tata Institute of Fundamental Research, Mumbai 400 005, India,; ^2^Université Libre de Bruxelles (ULB), ULB Institute of Neuroscience, B-6041 Gosselies, Belgium,; ^3^Centre of Excellence in Epigenetics, Indian Institute of Science, Education and Research, Pune 411 008, India, and; ^4^Department of Life Sciences, Sophia College for Women, Mumbai 400 026, India

**Keywords:** cell fate, glia, hippocampus, neuron

## Abstract

Regulation of the neuron–glia cell-fate switch is a critical step in the development of the CNS. Previously, we demonstrated that Lhx2 is a necessary and sufficient regulator of this process in the mouse hippocampal primordium, such that Lhx2 overexpression promotes neurogenesis and suppresses gliogenesis, whereas loss of Lhx2 has the opposite effect. We tested a series of transcription factors for their ability to mimic Lhx2 overexpression and suppress baseline gliogenesis, and also to compensate for loss of Lhx2 and suppress the resulting enhanced level of gliogenesis in the hippocampus. Here, we demonstrate a novel function of Dmrt5/Dmrta2 as a neurogenic factor in the developing hippocampus. We show that Dmrt5, as well as known neurogenic factors Neurog2 and Pax6, can each not only mimic *Lhx2* overexpression, but also can compensate for loss of Lhx2 to different extents. We further uncover a reciprocal regulatory relationship between Dmrt5 and Lhx2, such that each can compensate for loss of the other. Dmrt5 and Lhx2 also have opposing regulatory control on *Pax6* and *Neurog2*, indicating a complex bidirectionally regulated network that controls the neuron–glia cell-fate switch.

**SIGNIFICANCE STATEMENT** We identify Dmrt5 as a novel regulator of the neuron–glia cell-fate switch in the developing hippocampus. We demonstrate Dmrt5 to be neurogenic, and reciprocally regulated by Lhx2: loss of either factor promotes gliogenesis; overexpression of either factor suppresses gliogenesis and promotes neurogenesis; each can substitute for loss of the other. Furthermore, each factor has opposing effects on established neurogenic genes *Neurog2* and *Pax6*. Dmrt5 is known to suppress their expression, and we show that Lhx2 is required to maintain it. Our study reveals a complex regulatory network with bidirectional control of a fundamental feature of CNS development, the control of the production of neurons versus astroglia in the developing hippocampus.

Finally, we confirm that Lhx2 binds a highly conserved putative enhancer of *Dmrt5*, suggesting an evolutionarily conserved regulatory relationship between these factors. Our findings uncover a complex network that involves Lhx2, Dmrt5, Neurog2, and Pax6, and that ensures the appropriate amount and timing of neurogenesis and gliogenesis in the developing hippocampus.

## Introduction

One of the fundamental questions in neurodevelopment is how the balance between neurons and glia is regulated. In the CNS, neurons and astrocytes are thought to arise from common progenitors in a temporal sequence, such that neurogenesis precedes astrogliogenesis (Miller and Gauthier, 2007). How the timing of this transition, termed the “neuron–glia cell-fate switch,” is controlled, is a central question in building a functional nervous system.

Previously, we reported that Lhx2 overexpression in the developing hippocampus enhances and prolongs neurogenesis to generate neurons from progenitors that would otherwise give rise to astrocytes, whereas loss of Lhx2 causes premature astrogliogenesis. This role of Lhx2 was specific to the hippocampus, since loss of Lhx2 in the neocortical primordium did not enhance astrogliogenesis (Subramanian et al., 2011). In this study, we sought to identify potential downstream regulators of the neuron–glia cell-fate switch that may act as effectors of Lhx2 action. Using a candidate gene approach, we examined genes known to interact with Lhx2 or known to be neurogenic in other systems, such as the neocortex. Here, we uncover a new player in the regulation of the neuron–glia cell-fate switch in the hippocampus: Doublesex and mab-3-related transcription factor 5 (Dmrt5/Dmrta2), which functions in a mutually cross-regulatory network with Lhx2. We identify Neurog2 and Pax6, known to be repressed by Dmrt5 (Saulnier et al., 2013), as potential downstream targets of Lhx2. We demonstrate that all three genes—*Neurog2*, *Pax6*, and *Dmrt5*—are dependent on Lhx2 for their expression in the developing hippocampal primordium, suggesting that they may be direct or indirect targets of Lhx2. We show that each of these factors is able to partially or completely rescue the enhanced gliogenesis resulting from loss of Lhx2, indicating they function as part of a network downstream of Lhx2 to regulate this critical cell-fate decision. Finally, we demonstrate that Dmrt5 and Lhx2 can each rescue the enhanced gliogenesis that results from the loss of the other.

Dmrt5, a novel regulator of the neuron–glia cell-fate switch in the hippocampus, has not been previously reported to promote neurogenesis or suppress gliogenesis in any system. Our study reveals that Lhx2 binds a putative enhancer of *Dmrt5* in a region that is highly conserved across *Xenopus*, chick, mouse, and human. Thus, the reciprocal regulation between Lhx2 and Dmrt5 may be part of an evolutionarily conserved mechanism in the hippocampus that suppresses astrogliogenesis until neurogenesis is complete.

## Materials and Methods

### 

#### 

##### Mice.

All animal protocols were approved by the Institutional Animal Ethics Committee of the Tata Institute of Fundamental Research, Mumbai, India, according to regulations formulated by the India Committee for the Purpose of Control and Supervision of Experiments on Animals. For animal experiments performed at the Université Libre de Bruxelles (ULB), Institut de Biologie et de Médecine Moléculaires (IBMM), animal care followed institutional guidelines and the policies of the US National Institutes of Health.

The *Lhx2^lox/lox^*, *Emx1Cre^YL^* (Jin et al., 2000), and *Emx1Cre^KJ^* (Gorski et al., 2002) lines used in this study have been described previously (Shetty et al., 2013). The *Emx1Cre^YL^* line (Jin et al., 2000) was a gift from Yuging Li at the University of Florida College of Medicine. The *Emx1Cre^KJ^* line (Gorski et al., 2002) was a gift from Kevin R. Jones at the University of Colorado, Boulder. The *Lhx2^lox/lox^* line (Mangale et al., 2008) was a gift from Edwin S. Monuki at the University of California, Irvine. For generating embryos with a cortex-specific deletion of *Lhx2*, homozygous *Lhx2^lox/lox^* females were crossed to males of the genotype *Emx1Cre^YL^; Lhx2^lox/lox^*. The *Dmrt5^lox/lox^* line was a gift from David Zarkower and was generated as described by De Clercq et al. (2016). For generating a cortex-specific deletion of Dmrt5, homozygous *Dmrt5^lox/lox^* females were crossed to males of the genotype *Emx1Cre^KJ^; Dmrt5^lox/lox^*.

Timed pregnant female mice were obtained from the Tata Institute animal breeding facility and from the ULB, IBMM, animal breeding facility. Noon of the day the vaginal plug was observed was considered embryonic day (E) 0.5. Controls used for each experiment were age-matched littermates. Sexing is not possible by external observation at embryonic stages, so it is expected that the embryos used were a combination of both sexes. Embryos were killed by cervical dislocation in accordance with the guidelines prescribed by the Institutional Animal Ethics Committee.

##### *In situ* hybridization.

Digoxigenin (Dig)-labeled RNA probes were used for *in situ* hybridization (ISH). Dig-labeled nucleotide triphosphates were obtained from Roche and used to make riboprobes. *Emx1Cre^YL^ Lhx2^lox/lox^* control and mutant brains were sectioned (30 μm) using a freezing microtome. For section ISH of the *Emx1Cre^KJ^ Dmrt5^lox/lox^* control and mutant brains, 20 μm cryostat sections of 4% paraformaldehyde-fixed, 30% sucrose/PBS-infused tissue frozen in gelatin (7.5% gelatin, 15% sucrose/PBS) were used.

The microtome cut sections were mounted on Superfrost Plus slides (Erie Scientific). After fixing in 4% (w/v) paraformaldehyde, sections were washed with 1× PBS. The sections were then treated with proteinase K in Tris-EDTA buffer (1 μg/ml). Postfixation was done using 4% PFA and the sections were washed with 1× PBS. The sections were hybridized for 16 h at 70°C in buffer containing 50% (v/v) formamide, 5× SSC, and 1% (w/v) SDS. Stringent washes after hybridization were performed with solution X (50% formamide, 2× SSC, and 1% SDS) followed by 2× SSC and then 0.2× SSC. Overnight incubation at 4°C with anti-Dig antibody tagged with alkaline phosphatase (1:5000; Roche, catalog #12486523). Antibody was detected using substrate NBT/BCIP (Roche, 4-nitroblue tetrazolium chloride, catalog #70210625; 5-bromo-4-chloro-3-idolyl phosphate, catalog #70251721). Slides were counterstained with Fast Red (Sigma-Aldrich, N3020), coverslipped using DPX mountant, and imaged. ISH for each marker was performed in ≥3 biological replicates.

ISH for the cryostat cut sections were performed using antisense Dig-labeled ribropobes as described previously (Saulnier et al., 2013)

Plasmids used for generating probes were obtained from Grady Saunders, University of Texas M.D. Anderson Cancer Center (*Pax6*); Elizabeth Grove, University of Chicago (*Neurog2*); and Weiping Zhang, Second Military Medical University (*Zbtb20*). The *Dmrt5* probe was synthesized by linearizing EST AI592924 (GenBank).

Probes for *Lhx2* and *Prox1* were generated using PCR primers, the information for which is as follows (5′–3′): *Lhx2* forward: GATGTAGCTGCCCCCACGCC; *Lhx2* reverse: TGTGGAACAGCATCGCGGC; *Prox1* forward: ATGCAATTAACCCTCACTAAAGGGGCAGGCCTACTATGAGCCAG; *Prox1* reverse: ATGCTAATACGACTCACTATAGGGTTTGACCACCGTGTCCACAA.

##### *In utero* electroporation.

All procedures conducted followed the guidelines prescribed by the Institutional Animal Ethics Committee. Swiss mice obtained from the Tata Institute of Fundamental Research animal breeding facility were used for electroporation. E15 timed pregnant mice were anesthetized using either isoflurane (Forane, Abbott India) or a total of 2.5% (w/v) avertin [stock, 1 g/ml solution of 2,2,2-tribromoethanol (97%) in tert-amylalcohol (99+%); Sigma-Aldrich] in 0.9% saline was injected intraperitoneally (15 μl/g of body weight). The surgical procedure performed has been described previously (Subramanian et al., 2011). Using a fine-glass microcapillary, 3–4 μl plasmid DNA of concentration ∼2 μg/μl dissolved in nuclease free water and mixed with Fast Green dye was injected into the lateral ventricle of the embryos. For electroporation, a BTX CUY21 electroporator (40 V, five pulses, 50 ms pulse length, 1.0 s pulse interval) was used. Electric pulses were delivered using 5 mm paddle electrodes. The hippocampus was targeted by placing the positive electrode directed toward the medial side of the lateral ventricle in which the DNA was injected. The uterine horns were replaced and the incision was sewn with surgical sutures. Animals were kept on a 37°C warm plate for half an hour for postsurgical recovery. An oral suspension of meloxicam (Melonex, United Pharmacies) was mixed with the water in the feeding bottles of the dams (0.6 μl/ml) as an analgesic and given to the animals until 2 d after surgery. DNA construct pCAG-IRES2-EGFP was used as described by Subramanian et al. (2011). pACT2-r*Pax6*, a gift from Masaharu Sakai, encodes *Pax6* downstream of the β-actin promoter. This construct lacks a reporter. Therefore, electroporation was performed by mixing it with the *EGFP* construct in a 1:1 ratio.

##### Immunohistochemistry.

Primary antibodies used were as follows: rabbit anti-GFAP (1:200; Sigma-Aldrich, catalog #G9269; RRID: AB_477035) and biotinylated goat anti-GFP (1:400; Abcam, catalog #ab6658; RRID: AB_305631). Secondary antibodies used were as follows: streptavidin Alexa 488 (1:800; Invitrogen, catalog #S32354; RRID: AB_2315383) for GFP. Goat anti-rabbit antibody conjugated to Alexa 568 (1:400; Thermo Fisher Scientific, catalog #A11011; RRID: AB_143157) for GFAP. Tissue processing for immunohistochemistry was performed as described by Subramanian et al. (2011). Immunohistochemistry for calculating percentage astrocytes in electroporated brains was performed in three biological replicates.

##### *Ex utero* electroporation and dissociated culture.

Experiments using wild-type and *Lhx2^lox/lox^* embryos were performed at the Tata Institute of Fundamental Research (TIFR), Mumbai, and experiments using *Dmrt5^lox/lox^* embryos were performed at ULB, Brussels. Embryos were dissected out from the uterus, and the brains were removed and placed in sterile cold L-15 medium. Plasmid DNA at a concentration of 2 μg/μl (prepared using a Macherey-Nagel Maxi-prep kit) was injected into the ventricle of the brain. The brain was then electroporated on the medial side with five square pulses of 50 V of 50 ms duration, with a 1 s gap between each pulse, using a BTX Electro Square Porator ECM 830 electroporator and 3 mm paddle electrodes. Following this, the electroporated hemisphere was separated and the meninges were removed. The hippocampus was dissected from the electroporated hemisphere. The hippocampal explant was then maintained on a Millicell culture insert (Millipore, catalog #PICM03050) in Neurobasal medium containing B-27 supplement for 2 h in a 5% CO_2_ atmosphere. After 2 h, the explant was removed and treated with 0.25% trypsin, followed by treatment with trypsin-inhibitor solution and then dissociated by trituration. The dissociated cells were cultured on poly-d-lysine-coated (Sigma-Aldrich, catalog #P7280) coverslips in Neurobasal containing B-27 supplement for 5 d in a 5% CO_2_ atmosphere. The medium was changed every 2 d. For nonelectroporated wild-type cultures, the same protocol as above was used without the electroporation step.

DNA constructs *pCAG-IRES2-EGFP*, *CreGFP, Lhx2GFP* are as described in Subramanian et al., 2011. CBIG-Ngn2 was a gift from Jeffrey D. Macklis (Addgene plasmid #48708). It encodes *Neurog2* under the CAG promoter with a GFP reporter. For coelectroporation experiments, EGFP reporter was removed and the plasmid was then used for electroporation by mixing it with the *CreGFP* construct in a 1:1 ratio. *pEFXmDmrt5* (gift from Elizabeth Grove, University of Chicago) and *pACT2 Pax6* were mixed either with the *EGFP* construct or the *CreGFP* construct in a 1:1 ratio.

##### Immunostaining of dissociated cultures.

Primary antibodies used were as follows: biotinylated goat anti-GFP (1:400; Abcam, catalog #ab6658; RRID: AB_305631), rabbit anti-GFAP (1:200; Sigma-Aldrich, catalog #G9269; RRID: AB_477035), rabbit anti-GFAP (1:200; Dako, catalog #Z0334; RRID: AB_2100952), mouse GFAP (1:400; Sigma-Aldrich, catalog #G3893; RRID: AB_477010), rabbit β3-tubulin (1:500; Cell Signaling Technology, catalog #D65A4; RRID: AB_10691594), mouse β3-tubulin (1:500; Promega, catalog #G7128). Secondary antibodies used were as follows: streptavidin Alexa 488 (1:800; Invitrogen, catalog #S32354; RRID: AB_2315383) and fluorescein streptavidin (1:400; Vector Labs, Vector Sa-5001; RRID: AB_2336462) for GFP; goat anti-rabbit antibody conjugated to Alexa 568 (1:400; Molecular Probes, catalog #A11011; RRID: AB_143157), goat anti-rabbit antibody conjugated to Alexa 594 (1:400; Molecular Probes, catalog #A11012; RRID: AB_10562717), goat anti-rabbit antibody conjugated to Alexa 633 (1:400; Molecular Probes, catalog #A-21071; RRID: AB_10563600), and goat anti-mouse antibody conjugated to Alexa 568 (1:500; Molecular Probes, catalog #A11004; RRID: AB_2534072) for GFAP; and goat anti-rabbit antibody conjugated to Alexa 488 (1:500; Molecular Probes, catalog #A11008; RRID: AB_143165) and goat anti-mouse antibody conjugated to Alexa 647 (1:500; Molecular Probes, catalog #A-21235; RRID: AB_141693) for β3-tubulin.

The cultured cells were washed three times with cold PBS and fixed in 4% (w/v) PFA for 20 min at room temperature (RT). After fixation, the cells were kept in a quenching solution (75 mm ammonium chloride and 20 mm glycine in PBS) for 10 min. The cells were then kept in a block solution (10% FBS, 0.1% Triton X-100 in PBS) for 30 min at 37°C. After the blocking step, the cells were incubated overnight with block solution containing the primary antibody in the dilutions as mentioned above, at 4°C. After antibody incubation, the cells were given four washes of 5 min each with the blocking buffer, followed by incubation with the secondary antibody at the previously mentioned dilutions at 37°C for 2 h in dark. The cells were given four washes with blocking buffer, followed by two washes with PBS containing 0.1% Triton X-100. The cells were then stained with DAPI solution and mounted in glycerol containing antifade reagent (Invitrogen).

##### Experimental design and statistical analysis.

Animals were genotyped and assigned to control or experimental groups. Controls used for each experiment were age-matched littermates. GFP-expressing cells were scored from three different embryos per condition (three biological replicates). Colocalization with GFAP was determined by examining confocal images of the cells layer by layer and visually following all the processes of each GFP^+^ cell to identify regions of GFAP coexpression. All cells that displayed GFAP expression in one or more processes, or in the cell body, were counted as astroglia. A total of 300–400 cells were scored for each experiment on the wild-type and *Dmrt5^lox/lox^* backgrounds, and 200–300 cells for each experiment on the *Lhx2^lox/lox^* background. Statistical analysis was performed using the unpaired two-tailed Student's *t* test. Error bars represent SEM (*p ≤ 0.05; **p ≤ 0.001; ***p ≤ 0.0001; ns, not significant).

##### Imaging.

Bright-field images were taken using a Zeiss Axioplan 2+ microscope, Nikon Digital Sight DS-F12 camera, and Nikon NIS 4.0 imaging software. Images of immunohistochemistry and immunostaining of electroporated brains and dissociated cultures were obtained using a Zeiss LSM 5 Exciter–AxioImager M1 imaging system and Zeiss LSM710 imaging system. Image stacks were generated by scanning at intervals of 0.5–1 μm using filters of the appropriate wavelengths. The stacks were analyzed, merged, and projected using ImageJ software (RRID: SCR_003070) from the National Institutes of Health. Figure panels were prepared using Adobe Photoshop CS6. Figure 2*C*,*D* shows stitched composites from multiple confocal image frames.

##### Electrophoretic mobility shift assay.

Plasmid constructs encoding full-length Lhx2 protein, a truncated form that lacks the N-terminal Lim domain (Lhx2-ΔLim) or a homeodomain-deficient Lhx2 (Lhx2-ΔHD) have been described previously (Honda et al., 2012). The proteins were produced *in vitro* using the TNT coupled transcription-translation system (Promega). For the gel shift assay, double-stranded oligonucleotide probes containing the published Lhx2 binding site and the second putative site, in the 6.4 kb downstream region of *Dmrt5* (5′-AGTTGCCTAATTCCACTTTAATTGGAAAGG-3′), or their mutated versions (5′-AGTTGCCGCCGGCCACTTGCCGGGGAAAGG-3′), were generated by annealing complementary oligonucleotides and labeling them with [γ-^32^P] ATP and T4 polynucleotide kinase. Protein–DNA complexes were formed by incubation of 3 μl of *in vitro* translated protein with 50,000 cpm of the radiolabeled DNA probes for 20 min at RT in 20 μl of binding buffer (20 mm HEPES, pH 7.3, 60 mm KCl, 1 mm DTT, 1 mm MgCl_2_, 0.1 mm EDTA, 10% glycerol, 0.5 mg/ml BSA) containing 1 μg of poly(deoxyinoinic-deoxycytidylic) acid sodium salt. The DNA–protein complex was resolved on a 6% native PAGE in Tris-glycine 1× buffer. The gel was fixed in 10% acetic acid and 10% methanol, and then dried. The complex formation was assessed by autoradiography. Electrophoretic mobility shift assay (EMSA) was performed in two biological replicates.

##### Chromatin immunoprecipitation quantitative PCR.

Embryonic brains from E12 embryos and E15 embryos were harvested and the hippocampal tissue was isolated in cold 0.5% glucose in PBS with 1× protease inhibitor mixture (Sigma-Aldrich). The tissue was cross-linked immediately after harvesting with 1% formaldehyde (Thermo Fisher Scientific). To obtain chromatin, the cells were lysed and a Covaris S220 sonicator was used for 15 cycles (E12 tissue) or 18 cycles (E15 tissue) of 60 s ON and 30 s OFF (5% duty cycle, 2 intensity, and 200 cycles per burst) to get chromatin within the size range of 100–500 bp. Ten micrograms of chromatin and 2 μg of antibody were used per immunoprecipitation (IP). The following antibodies were used for chromatin IP (ChIP): goat α-Lhx2 (Santa Cruz Biotechnology, SC19344; RRID: AB_2135660) and goat IgG (Bangalore Genei). The protein–DNA complex was pulled down using Protein A/G Magnetic Beads (Dynabeads, Invitrogen). The IP DNA was purified using phenol-chloroform-isoamyl alcohol (Ambion). Fold enrichment over control IgG was assessed by performing ChIP quantitative PCR (qPCR) using the SYBR Green master mix (Roche) and primers specific for the Lhx2 binding region in the *Dmrt5* genomic locus and for a control genomic region. ChIP qPCRs were done in technical duplicates and three independent experiments (biological replicates) were performed. For statistical analysis, independent experiments were used to calculate average, SEM, and significance values.

The primers used for ChIP qPCR are as follows (5′ to 3′): Lhx2 binding region on *Dmrt5* forward, GGCGGTGAAACTTAATAGCAGG; Lhx2 binding region on *Dmrt5* reverse, CTCTTCGTCACCCTCACACT; control genomic region forward, GGGTCACTGAGGCAAAAATC; control genomic region reverse, GCCTATCACCTGCAGGATTC.

##### ECR Browser genome analysis for conserved sequences.

The ECR (evolutionary conserved regions) Browser (Ovcharenko et al., 2004) on-line graphical interface was used for analyzing the conserved genomic regions in the *Dmrt5* locus spanning 50 kb across the transcription start site. The default settings on the ECR Browser was used for the analysis.

For the sequence alignment of the Lhx2 binding sites in mouse, human, and chick, ClustalW was used and the following genome assemblies were used for the analysis: human, GRCh37/hg19 assembly; mouse, NCBI37/mm9 assembly; chick, International Chicken Genome Sequencing Consortium Gallus_gallus-4.0/galGal4 assembly.

## Results

**Figure 1.**
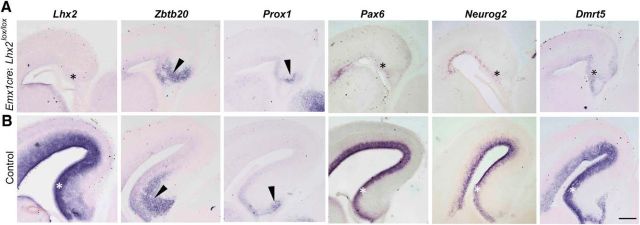
*Pax6*, *Neurog2*, and *Dmrt5* require Lhx2 for normal expression in the embryonic hippocampus. ***A***, ***B***, *EmxCre; Lhx2^lox/lox^* brains at E15 display shrunken hippocampal primordia that express *Zbtb20* and *Prox1* (arrowheads), indicating that the hippocampus is specified. *Pax6*, *Neurog2*, and *Dmrt5*, normally expressed in the hippocampal ventricular zone (***B***, white asterisks), are greatly reduced upon loss of *Lhx2* (***A***, black asterisks). Scale bar, 200 μm.

*Lhx2* is normally expressed in the dorsal telencephalic ventricular zone at E15 (Fig. 1; Bulchand et al., 2003). We used *Emx1Cre* as the driver to delete *Lhx2* in the dorsal telencephalon, and examined the hippocampal primordium at E15. Though the hippocampal primordium is considerably smaller in the mutant than in control embryos, the expression of molecular markers *Zbtb20* and *Prox1* indicate that hippocampal identity has been specified (Fig. 1). Since Lhx2 has been previously demonstrated to be a necessary and sufficient regulator of the neuron–glia cell-fate switch in the developing hippocampus, we examined candidate genes as potential Lhx2 targets in this structure. Of these, three genes normally expressed in the hippocampal ventricular zone were barely detectable upon loss of *Lhx2* (Fig. 1): *Pax6*, a known Lhx2 target (Shetty et al., 2013) and also known for its neurogenic properties (Heins et al., 2002); *Neurog2*, an established neurogenic gene (Nieto et al., 2001); and *Dmrt5*, which we examined because it was demonstrated to be an upstream regulator of *Lhx2* (Saulnier et al., 2013). The results indicate that *Dmrt5* has a bidirectional regulatory relationship with *Lhx2*, since its expression is drastically reduced in the ventricular zone upon loss of Lhx2 (Fig. 1).

**Figure 2.**
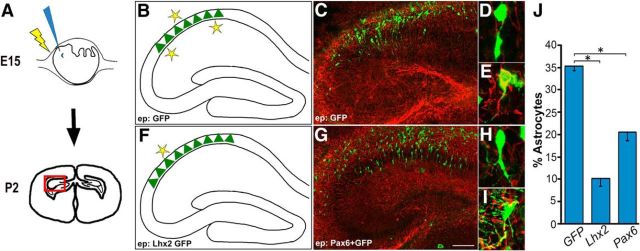
Overexpression of *Pax6* enhances neurogenesis and suppresses astrogliogenesis in the developing hippocampus. ***A***, Schematic showing *in utero* electroporation at E15 and harvesting the brains at postnatal day 2 (P2). ***B***, ***F***, Schematic summarizing the results of *Lhx2* overexpression (***F***) by *in utero* electroporation at E15, which results in enhanced neurogenesis and reduced gliogenesis, compared with control GFP electroporation (***B***). ***F***, ***G***, *Pax6* overexpression (***G***) appears to mimic *Lhx2* overexpression (***F***). ***C–E***, ***G–I***, High-magnification images displaying GFAP (red) and GFP (green) expression reveals neurons (***D***, ***H***) and astrocytes (***E***, ***I***) alongside their corresponding low-magnification images (***C***, ***G***). ***J***, Bar graph showing the percentage of GFP-expressing cells that coexpress GFAP. Control GFP electroporation results in 35% astrogliogenesis, which is suppressed to 10% upon *Lhx2* overexpression (Subramanian et al., 2011) and 20% upon *Pax6* overexpression. Images in ***C*** and ***G*** are stitched composites of multiple frames. Scale bars: ***C***, ***G***, 100 μm; ***D***, ***E***, ***H***, ***I***, 15 μm. *p < 0.05.

Lhx2 overexpression is capable of suppressing gliogenesis at E15 and E17, as demonstrated using *in utero* electroporation of a construct encoding full-length Lhx2 with a GFP reporter (Fig. 2*A*,*B*,*F*; Subramanian et al., 2011). In these assays, the number of electroporated (GFP-expressing) cells that also coexpressed astroglial marker GFAP was scored. Lhx2 overexpression brought the level of astrogliogenesis at E15 down from 35 to 10% (Fig. 2*J*; data replotted from Subramanian et al., 2011). In the same study, we also demonstrated the neuronal identity of Lhx2-overexpressing cells by their expression of β-tubulin, their lack of expression of GFAP, and their ability to extend axons into the fimbria. Furthermore, we confirmed that GFAP-expressing cells produced by loss of Lhx2 were astroglia, since they also expressed AldoC, and did not express Olig2 (Subramanian et al., 2011). GFAP upregulation in astrocytes is a result of activation of the JAK-STAT pathway and the action of progliogenic factor Nfia (Bonni et al., 1997; Cebolla and Vallejo, 2006). Lhx2 is able to suppress Nfia-induced astrogliogenesis as well as GFAP upregulation (Subramanian et al., 2011). Therefore, in the present study, we scored the percentage of GFP-electroporated cells that coexpressed GFAP as a measure of the level of astrogliogenesis in progenitors under different experimental conditions. This is also consistent with previous studies that examined the neuron–glia cell-fate switch in the neocortex (Sun et al., 2001; Fan et al., 2005; Hirabayashi et al., 2009).

Since *Pax6* is a known Lhx2 target, we first tested *Pax6* overexpression in wild-type embryos at E15, and examined the brains at postnatal day 2. Only 20% of the electroporated cells coexpressed GFAP, indicating that Pax6 is able to partially mimic Lhx2 in suppressing gliogenesis (Fig. 2*G–J*).

**Figure 3.**
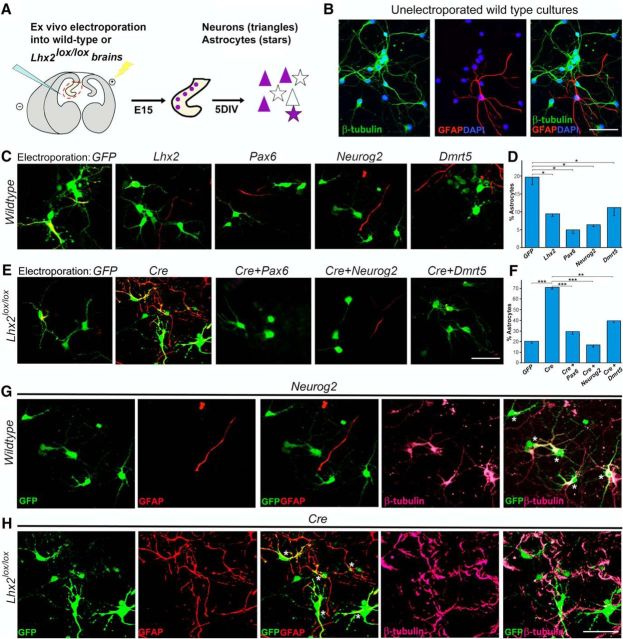
*Pax6*, *Neurog2*, or *Dmrt5* can mimic *Lhx2* overexpression and can rescue the enhanced astrogliogenesis resulting from loss of Lhx2. ***A***, Diagram illustrating *ex vivo* electroporation followed by dissociated cell culture. At E15, electroporated progenitors (purple) produce more neurons (purple triangles) than glia (purple stars), and nonelectroporated cells of both types (clear triangles and stars) are also seen. ***B***, A nonelectroporated dissociated cell culture from a wild-type embryo displays β-tubulin-expressing neurons (green) and an occasional GFAP-expressing astrocyte (red). DAPI staining (blue) identifies the nuclei of all the cells in the field. ***C***, ***D***, Dissociated cell cultures from wild-type E15 embryonic hippocampi electroporated *ex vivo* with constructs encoding *GFP*, *Lhx2*, *Pax6*, *Neurog2*, and *Dmrt5*. ***E***, ***F***, Dissociated cell cultures from E15 *Lhx2^lox/lox^* hippocampi electroporated *ex vivo* with constructs encoding *GFP*, *CreGFP*, *CreGFP*+*Pax6*, *CreGFP*+*Neurog2*, or *CreGFP*+*Dmrt5*. After 5 d *in vitro*, the percentage of electroporated (GFP-expressing, green) cells that also expressed astrocyte marker GFAP (red) was scored. ***D***, Quantification of the results reveals 20% of the cells to be astrocytes following control *GFP* electroporation into wild-type hippocampi, which decreases upon electroporation of constructs encoding neurogenic factors to 9% (*Lhx2*), 5% (*Pax6*), 6% (*Neurog2*), and 11% (*Dmrt5*). ***F***, In the *Lhx2^lox/lox^* background, quantification of the results reveals 21% of the cells to be astrocytes upon control *GFP* electroporation, which increases to 71% upon loss of *Lhx2* as a result of *CreGFP* electroporation. Coelectroporation of constructs encoding neurogenic factors together with *CreGFP* restores the percentage astrocytes to 30% (*Pax6*), 17% (*Neurog2*), and 39% (*Dmrt5*). ***G***, ***H***, Individual GFP (green), GFAP (red), and β-tubulin (pink) channels as well as GFP–GFAP and GFP–β-tubulin overlays for *Neurog2* electroporation in wild-type hippocampi (***G***) and Cre electroporation in *Lhx2^lox/lox^* hippocampi (***H***). Asterisks indicate GFP–β-tubulin-coexpressing neurons (***G***) or GFP–GFAP-coexpressing astrocytes (***H***). Scale bars: 50 μm. *p < 0.05, **p,0.001, ***p < 0.0001.

We sought to establish a more efficient *in vitro* assay for regulation of the neuron–glia cell-fate switch in hippocampal progenitors (Fig. 3*A*). First, we prepared dissociated cultures of E15 wild-type hippocampal tissue and confirmed that after 5 d *in vitro* all the cells express either neuronal (β-tubulin) or glial (GFAP) markers (Fig. 3*B*). Then, we added the step of *ex vivo* electroporation, in which DNA is injected into the telencephalic ventricles, and electroporation of the intact brain is performed in a Petri dish. In such a preparation, the progenitors get transfected in a manner similar to *in utero* electroporation. The hippocampal primordium is then isolated and the cells maintained in dissociated cell culture (Fig. 3*A*). The advantage of *ex vivo* electroporation is that the hippocampus, being a more difficult structure to target than the lateral neocortex, is easily electroporated, and every single embryo in the litter can be used. Furthermore, since cells from a single embryo are used for one coverslip, multiple experiments using different constructs can be performed in parallel from a single litter. Therefore, this system has considerable advantages over *in utero* electroporation. We first tested whether *ex vivo* electroporation followed by dissociated cell culture recapitulates the findings we demonstrated using *in utero* electroporation (Subramanian et al., 2011). We found the baseline level of gliogenesis to be 20% in the dissociated cultures, which is suppressed to 9% by *Lhx2* overexpression and to 5% by *Pax6* overexpression. This indicates that the *in vitro* assay reproduces the functional effects of overexpression of both genes (Fig. 3*B*,*C*). We then tested *Neurog2* and *Dmrt5* by overexpressing them in wild-type embryos. Both genes suppressed baseline astrogliogenesis to 6 and 11% respectively. These results confirm the function of Neurog2 as a strong regulator of the neuron–glia cell-fate switch in the hippocampus, and demonstrate for the first time a similar function for Dmrt5 (Fig. 3*B*,*C*).

The striking decrease in *Pax6*, *Neurog2*, and *Dmrt5* expression upon loss of Lhx2 (Fig. 1*A*) and their ability to suppress astrogliogenesis when overexpressed (Figs. 2*G,J*, 3*C*,*D*) suggested that they might mediate the role of Lhx2 in this process. Therefore, we tested whether each of these genes can rescue the progliogenic effects of loss of Lhx2. In the *Lhx2^lox/lox^* background, baseline gliogenesis at E15 is 21%. Loss of Lhx2 by electroporation of *CreGFP* increases this to 71% (Fig. 3*E*,*F*), consistent with our previously reported results (Subramanian et al., 2011). When we coelectroporated either *Pax6*, or *Neurog2*, or *Dmrt5* together with *CreGFP*, we found that each of these genes is able to rescue neurogenesis to 30, 17, and 39% respectively (Fig. 3*E*,*F*). For the most gliogenic condition (*Cre* electroporation in *Lhx2^lox/lox^* embryos) and one of the most neurogenic conditions (*Neurog2* in wild-type embryos), we also examined β-tubulin expression to test for neuronal identity. As expected, the majority of *Neurog2* electroporated cells coexpressed β-tubulin indicating their neuronal identity (Fig. 3*G*), whereas cells that lost Lhx2 as a result of *Cre* electroporation did not coexpress β-tubulin (Fig. 3*H*).

Together, the results indicate that Pax6, Neurog2, and Dmrt5 are each capable of substituting for Lhx2 to different extents, and are therefore part of a network over which Lhx2 functions to regulate the neuron–glia cell-fate switch.

**Figure 4.**
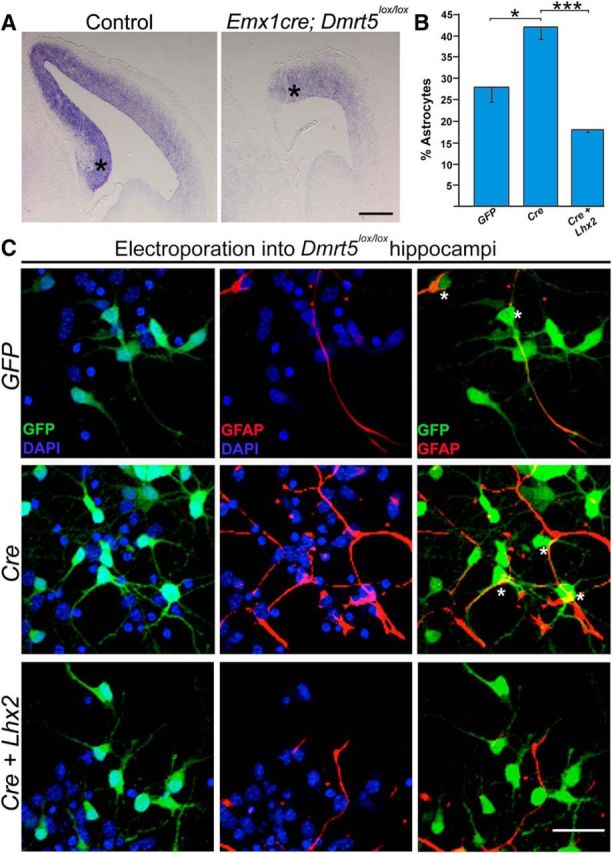
*Lhx2* can rescue the enhanced astrogliogenesis resulting from loss of Dmrt5. ***A***, *Lhx2* expression is decreased in *EmxCre; Dmrt5^lox/lox^* brains compared with controls. ***B***, ***C***, E15 embryonic hippocampi from *Dmrt5^lox/lox^* embryos were electroporated *ex vivo* with constructs encoding *GFP*, *CreGFP*, and *CreGFP*+*Lhx2*. After 5 d *in vitro*, the percentage of electroporated (GFP-expressing, green) cells that also expressed astrocyte marker GFAP (red) was scored. ***B***, Quantification of the results shows 28% of the cells to be astrocytes upon control *GFP* electroporation, which increased to 42% upon loss of *Dmrt5* as a result of *CreGFP* electroporation, and decreased to 18% upon coelectroporation of *Lhx2* together with *CreGFP*. ***C***, Individual GFP (green) + DAPI (blue) and GFAP (red) + DAPI (blue) channels, as well as GFP–GFAP overlays. DAPI staining (blue) identifies the nuclei of all the cells in the field. Asterisks indicate GFP–GFAP-coexpressing astrocytes. Scale bars: ***A***, 500 μm; ***C***, 30 μm. *p < 0.05, ***p < 0.0001.

Dmrt5 was previously shown to be a potential upstream regulator of *Lhx2*, and *Lhx2* expression levels decrease upon loss of *Dmrt5* (Saulnier et al., 2013; De Clercq et al., 2016; Fig. 4*A*). We tested whether loss of Dmrt5 is gliogenic by electroporating *CreGFP* into *Dmrt5^lox/lox^* hippocampi, and found that the baseline level of 28% gliogenesis is increased to 42%, demonstrating that Dmrt5, like Lhx2, is required to suppress premature astrogliogenesis. Upon coelectroporation of *Lhx2* with *CreGFP*, this enhanced gliogenesis is not only rescued, but also suppressed to 18%, below baseline levels (Fig. 4*B*,*C*). Together, the results suggest a reciprocally regulatory relationship between Dmrt5 and Lhx2.

**Figure 5.**
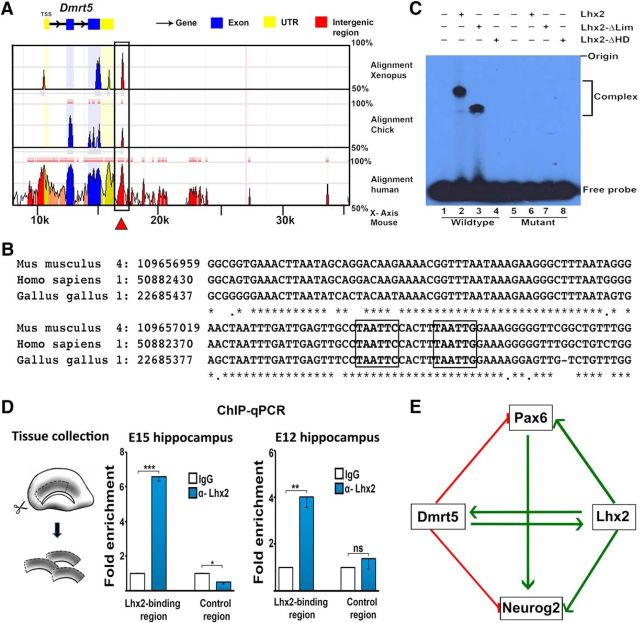
Lhx2 directly binds to the *Dmrt5* locus within an evolutionarily conserved distal regulatory element. ***A***, ***B***, ECR Browser view of the genomic region spanning 50 kb around the *Dmrt5* locus. The Lhx2 binding region lies within a region in the 3′ UTR of *Dmrt5* (***A***, red arrow) that is conserved across *Xenopus*, chick, and human. The Lhx2 binding site sequence reported in the literature (Folgueras et al., 2013) was identified in an element 6.4 kb downstream of *Dmrt5*. ***C***, DNA EMSA was performed as mentioned in Materials and Methods. Radiolabeled oligonucleotides containing the wild-type sequence at the Lhx2 binding site and the putative binding site, or oligonucleotides mutated at both sites, were incubated with no protein (Lanes 1 and 5), or with *in vitro* synthesized full- length Lhx2 (Lane 2 and 6), or with Lhx2-ΔLim (Lanes 3 and 7), or with Lhx2-ΔHD (Lanes 4 and 8). Incubation of the wild-type oligonucleotide with full-length Lhx2 (Lane 2) or with Lhx2-ΔLim (Lane 3) each resulted in retarded bands, whereas incubation with Lhx2-ΔHD (Lane 4) or with no protein (Lane 1) did not lead to gel retardation. EMSA with the oligonucleotide containing the mutated sites yielded no gel retardation with any protein (Lanes 5–8). ***D***, Diagram illustrating the tissue collection from E15 and E12 embryonic hippocampi for ChIP followed by qPCR. In chromatin from both E15 and E12 tissue, Lhx2 displays significant enrichment at its binding site on the Dmrt5 locus compared with a control genomic region. ***E***, A regulatory network of genetic interactions in the developing hippocampus. Lhx2 and Dmrt5 reciprocally regulate each other (Saulnier et al., 2013; De Clercq et al., 2016; this study). Lhx2 positively regulates *Neurog2* and *Pax6* (Shetty et al., 2013; this study), whereas Dmrt5 negatively regulates these factors (Saulnier et al., 2013). *Neurog2* is also regulated by Pax6 (Scardigli et al., 2003). **p < 0.001, ***p < 0.0001.

Since Dmrt5 is a novel player in the regulation of the neuron–glia cell-fate switch, we explored its regulatory relationship with Lhx2 further. No recognition sequence for Dmrt5 has been identified *in vivo*, but the Lhx2 binding site consensus sequence has been identified in several systems, including the developing forebrain (Marcos-Mondéjar et al., 2012; Muralidharan et al., 2017). Therefore, we examined a 50 kb region spanning the *Dmrt5* locus and identified the published Lhx2 binding site sequence (TAATTG; Folgueras et al., 2013) and a second putative binding site (TAATTC) residing in the intergenic region of the *Dmrt5* locus 6.4 kb downstream of the transcription start site. We then performed genomic sequence alignment across species, examining a 50 kb region spanning the *Dmrt5* locus, to assess whether intergenic regions around the *Dmrt5* locus show evolutionary conservation. Indeed, examination of *Xenopus*, chick, mouse, and human genomic sequences reveals the 6.4 kb downstream region we identified, containing the published Lhx2 binding site and the second putative binding site, to be highly conserved in the intergenic regions of the *Dmrt5* locus (Fig. 5*A*,*B*).

We performed an EMSA to test for direct binding of Lhx2 to a fragment spanning both these sites (Fig. 5*C*). The ability of Lhx2 to bind to these sites was tested using three forms of the Lhx2 protein produced in rabbit reticulocyte lysate, namely a full-length Lhx2 protein, a truncated form that lacks the N-terminal Lim domain (Lhx2-ΔLim), and a homeodomain-deficient Lhx2 (Lhx2-ΔHD). While the reticulocyte lysate alone resulted in no retarded bands, a distinct mobility shift was observed with the oligonucleotide containing the wild-type sequence at both sites. This binding was not detected when both sites were mutated (Fig. 5*C*, compare Lanes 2, 6). The Lhx2-ΔLim, but not the Lhx2-ΔHD also formed a complex with the oligonucleotide containing the wild-type sequence at both sites (Fig. 5*C*, compare Lanes 3, 4) indicating the requirement of the homeodomain region of Lhx2 for its sequence-specific DNA-binding activity. Together, these data indicate that Lhx2 binds directly to the *Dmrt5* locus at the binding site(s) we have identified.

We further tested whether Lhx2 occupies the *Dmrt5* locus *in vivo* by performing ChIP followed by qPCR using primers specific for the Lhx2 binding region, and for a control region lacking the Lhx2 binding site. We found enrichment of Lhx2 occupancy at the Lhx2 binding region in chromatin isolated from E15 as well as E12 embryonic hippocampal tissue (Fig. 5*D*). Together, these data demonstrate that Lhx2 displays continued occupancy on the *Dmrt5* locus starting from the early stages and up to the peak of hippocampal neurogenesis.

## Discussion

The mechanisms by which progenitors in the CNS produce first neurons and then glia have been a subject of great interest for decades, and this cell-fate switch has been established in multiple structures, including the neocortex (Temple, 2001; Sun et al., 2001; Fan et al., 2005; He et al., 2005; Hirabayashi et al., 2009; Namihira et al., 2009), the striatum (Reynolds et al., 1992), the retina (Turner and Cepko, 1987), and the spinal cord (Deneen et al., 2006). In the hippocampus, we reported Lhx2 to be a necessary and sufficient regulator of this cell-fate switch (Subramanian et al., 2011). In the present study, we identify a cross-regulatory network of transcription factors that interact with Lhx2 to execute this function. Loss of Lhx2 using a dorsal telencephalon-specific driver revealed known neurogenic genes *Pax6* and *Neurog2*, as well as a novel target *Dmrt5*, as potential effectors of Lhx2 function in regulating the neuron–glia cell-fate switch. Each of these transcription factors can promote neurogenesis and suppress gliogenesis in wild-type hippocampal progenitors, as well as progenitors derived from *Lhx2* mutant hippocampi. Each of these factors is positively regulated by Lhx2. In contrast, Dmrt5 represses *Pax6* expression via direct or indirect mechanisms (Saulnier et al., 2013; De Clercq et al., 2016) and suppresses *Neurog2* possibly via upregulation of Hes1 (Young et al., 2017). In addition, *Neurog2* is a direct target of Pax6 (Scardigli et al., 2003). Lhx2 and Dmrt5 display reciprocal regulation (Saulnier et al., 2013; De Clercq et al., 2016; this study). These interactions, summarized in Figure 5*E*, present a transcriptional network for the control of hippocampal neuron–glia cell fate.

*Neurog2* is part of a family of basic helix-loop-helix genes, including *Neurog1*, *Math1*, and *Mash1*, that have been implicated in regulation of neuronal differentiation in the developing CNS (Ben-Arie et al., 1997; Fode et al., 2000; Nieto et al., 2001; Sun et al., 2001). In *Neurog2/Mash1* double-mutant cortices, progenitors fail to take on a neuronal fate and instead become astrocytes (Nieto et al., 2001). Neurog1 is a well characterized regulator of the neuron–glia cell-fate switch: it promotes neurogenesis directly, and suppresses gliogenesis by sequestering CBP/P300 from Stat3, a progliogenic molecule (Sun et al., 2001). Like Neurog1, Neurog2 also interacts with CBP/p300 and this results in the activation of spinal motor neuron genes during development (Lee et al., 2009). Pax6, a direct regulator of Neurog2 (Scardigli et al., 2003), is itself known to promote neurogenesis in development (Heins et al., 2002) and also in the adult hippocampal neurogenic system (Klempin et al., 2012). A role for Pax6 in suppressing gliogenesis has not yet been reported, however, so understanding how overexpression of Pax6 reduces the percentage of glia in our system raises intriguing mechanistic questions.

*Neurog2* and *Pax6* are each under positive regulation by Lhx2 (Shetty et al., 2013; this study) and, interestingly, negative regulation by Dmrt5 since their expression in the dorsal telencephalon is increased upon loss of Dmrt5 (Saulnier et al., 2013; De Clercq et al., 2016; Young et al., 2017). This may explain why loss of Dmrt5 results in a smaller enhancement of gliogenesis than loss of Lhx2, since Dmrt5 and Lhx2 have opposite effects on the expression of neurogenic genes *Neurog2* and *Pax6*. The increased expression of these factors may counteract the gliogenic effect of loss of Dmrt5.

The reciprocal regulation between *Dmrt5* and *Lhx2* makes these interesting candidates to examine, and explains the many parallels between their known functions. Both genes are expressed in the telencephalic ventricular zone throughout the period of neurogenesis in a high-caudomedial to low-rostrolateral gradient (Nakagawa et al., 1999; Saulnier et al., 2013). Both genes are necessary for a normal cortical hem, which is expanded in the absence of Lhx2 and missing in the absence of Dmrt5. The hippocampus is lost in the *Dmrt5*-null and *Lhx2*-null embryos (Bulchand et al., 2001; Konno et al., 2012; Saulnier et al., 2013). Deletion of either *Dmrt5* or *Lhx2* after hem formation results in disrupted cortical arealization (Zembrzycki et al., 2015; De Clercq et al., 2016). The hippocampus is also reduced in size, indicating that both genes play a direct role in hippocampus development independent of their roles in the hem (Subramanian et al., 2011; De Clercq et al., 2016; this study). Loss of any one of them is sufficient to increase the percentage of glia arising from hippocampal progenitors, and their overexpression promotes neurogenesis (Subramanian et al., 2011; this study). How Dmrt5 regulates *Lhx2* is not yet understood. Yet, it is clear that maintenance of the high endogenous levels of *Lhx2* seen in the hippocampal primordium requires Dmrt5. In the absence of Dmrt5, the lowered level of *Lhx2* is apparently inadequate to suppress astrogliogenesis. However, *Lhx2* overexpression is sufficient to compensate for Dmrt5 loss of function, and brings astrogliogenesis to levels below the baseline, indicative of Lhx2 acting via multiple effectors to suppress gliogenesis and promote neurogenesis in the hippocampal primordium.

Our study reveals that Lhx2 and Dmrt5 function in a complex regulatory network with the intriguing feature that they appear to have opposite effects on two key factors, *Neurog2* and *Pax6*. This reinforcing and counter-balancing set of controls is indicative of a finely tuned bidirectionally regulated network, and motivates a full-scale exploration of other common targets of Dmrt5 and Lhx2, both of which appear to be ancient players in controlling fundamental features of forebrain development. In zebrafish, Dmrt5 regulates neurogenesis acting via Neurog1 (Yoshizawa et al., 2011) and Lhx2 is thought to mediate the proliferative function of Six3 in the forebrain (Ando et al., 2005). Our finding of a conserved putative enhancer in the *Dmrt5* locus that contains bonafide Lhx2 binding site(s) strengthens the idea of an evolutionarily conserved interaction between these two molecules in regulating the development of the forebrain and, in particular, the fundamental process of controlling the production of appropriate numbers of neurons and glia from common neuroepithelial progenitors.
